# The impact of 3D printed models on spatial orientation in echocardiography teaching

**DOI:** 10.1186/s12909-022-03242-9

**Published:** 2022-03-16

**Authors:** Christoph Salewski, Attila Nemeth, Rodrigo Sandoval Boburg, Rafal Berger, Hasan Hamdoun, Hannes Frenz, Sebastian Spintzyk, Julia Kelley Hahn, Christian Schlensak, Tobias Krüger

**Affiliations:** 1 Department of Thoracic and Cardiovascular Surgery, University Medical Center Tübingen, Tübingen, Germany; 2 Section for Medical Material Science and Technology at the Department of Thoracic and Cardiovascular Surgery, University Medical Center Tübingen, Tübingen, Germany

**Keywords:** Echocardiography, Cardiac surgery, Cardiology, 3D printing, Teaching echocardiography

## Abstract

**Purpose:**

During our transthoracic echocardiography (TTE) courses, medical students showed difficulty in spatial orientation. We implemented the use of 3D printed cardiac models of standard TTE views PLAX, PSAX, and A4C and assessed their efficacy in TTE-teaching.

**Methods:**

One hundred fifty-three participants were split into two groups. A pre-test-retest of anatomy, 2D -, and 3D orientation was conducted. The intervention group (*n* = 77) was taught using 3D models; the control group (*n* = 76) without. Both were comparable with respect to baseline parameters. Besides test-scores, a Likert scale recorded experiences, difficulties, and evaluation of teaching instruments.

**Results:**

From the 153 students evaluated, 123 improved, 20 did worse, and ten achieved the same result after the course. The median overall pre-test score was 29 of 41 points, and the retest score was 35 (*p* < 0.001). However, the intervention group taught with the 3D models, scored significantly better overall (*p* = 0.016), and in 2D-thinking (*p* = 0.002) and visual thinking (*p* = 0.006) subtests. A backward multivariate linear regression model revealed that the 3D models are a strong individual predictor of an excellent visual thinking score. In addition, our study showed that students with difficulty in visual thinking benefited considerably from the 3D models.

**Conclusion:**

Students taught using the 3D models significantly improved when compared with conventional teaching. Students regarded the provided models as most helpful in their learning process. We advocate the implementation of 3D-printed heart models featuring the standard views for teaching echocardiography. These findings may be transferable to other evidence based medical and surgical teaching interventions.

## Highlights

• Medical students present difficulty with spatial orientation in echocardiography.

• We asked for anatomical structures, 2D orientation, and 3D orientation.

• We used 3D printed models for teaching standard echocardiographic views.

• The 3D group (*n* = 77) performed significantly better than the control group (*n* = 76).

• We advocate 3D printed models for the teaching of echocardiography standard views.

## Introduction

Transthoracic echocardiography (TTE) is an essential clinical skill which is a routine part of cardiac surgery and cardiology resident training. However, also medical students often express an interest in this topic and request voluntary hands-on courses. Echocardiography offers an enhanced understanding of cardiac anatomy and physiology. Accordingly, the new German national competence based catalogue of learning objectives (NKLM) for medical schools suggests sonography as a skill, students should be exposed to during their studies [13].

Upon this request, we offered our first voluntary echocardiography curriculum in 2017; and the course proved to be very popular. The content of our course adhered to the CME certified education “Basic transthoracic echocardiographic skills in cardiac surgery patients [9]” and followed the standard best practice procedures for TTE training of national societies [5].

During the first 3 years of the program, students frequently reported problems in spatial intracardial orientation, which hampered image interpretation and the learning progress. To overcome this by didactical means, 3D printed cardiac models were introduced to this course.

Didactic advantages for the use of 3D models have been described in anatomical teaching [1], additionally, 3D models have been successfully used in cardiac surgery training [10] and procedure planning [17]. It therefore appears plausible to transfer the concept to echocardiographic training. Surely, 3D Models have been used earlier for TTE-teaching, but the didactic effects are poorly studied: in the only scientific evaluation we are aware of, no didactic advantages have been demonstrated for the 3D-model use [15].

Due to changes of the curriculum secondary to the COVID-19 restrictions, our voluntary course became obligatory for an entire semester cohort following strict hygienic standards. This allowed for us to cross-sectionally teach and study an entire semester-cohort in a highly standardized manner without bias to previous knowledge or special interests in this topic. Complementarily, the department of internal medicine has established an complementary sonographic curriculum [2].

The objective of this project was to study the didactical impact of 3D printed models of standard echocardiographic views on the students’ learning success during a teaching intervention in a controlled trial.

## Methods

### Study design

The study was designed as a unicentral, prospective, controlled, unblinded, cross-sectional trial, extensively comparing two equally sized groups of medical students of a complete, unselected semester-cohort for learning success in TTE with or without the usage of 3D models. Participation in the study was voluntary and subjects submitted their written informed consent. We enrolled 195 medical students in their 9th semester.

### The course

**Fig. 1:**
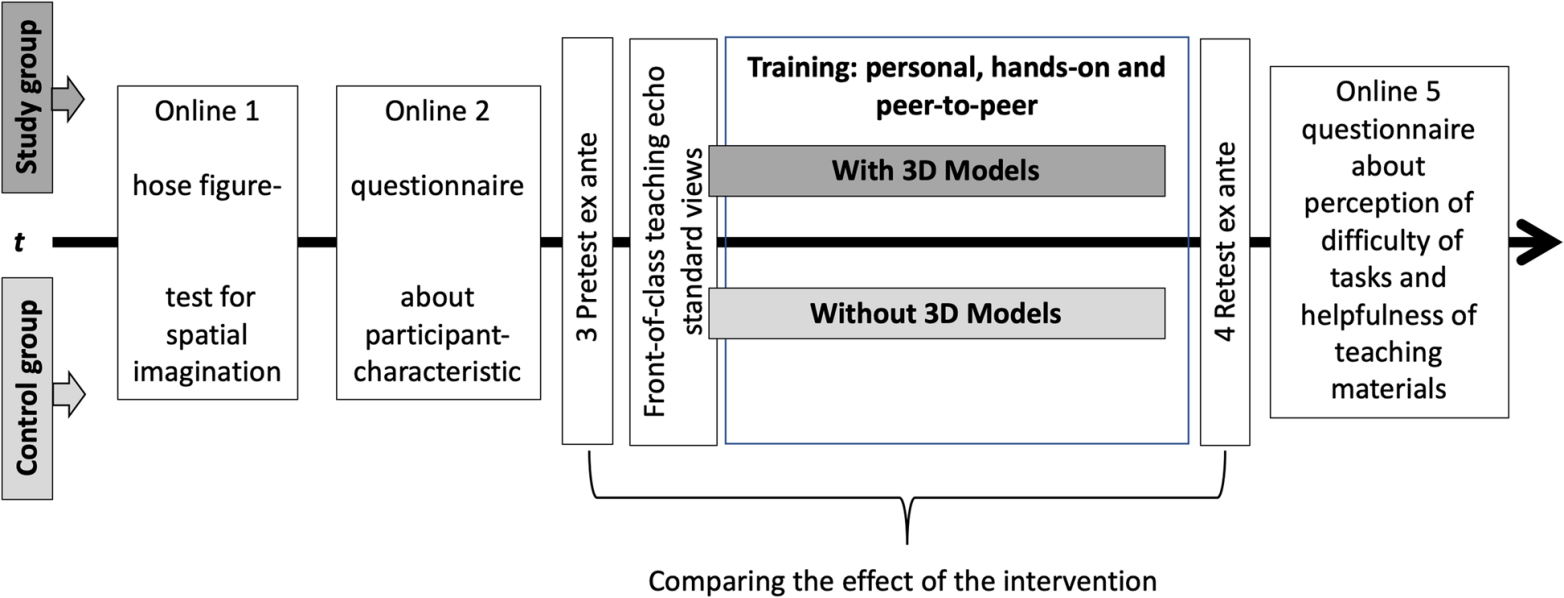
Study course. The study group and the control group participated in online courses and practical courses. The intervention took place during the hands-on experience. Later both groups filled in a questionnaire about difficulty and helpfulness

The educational objective was to learn, understand, and reproduce three basic standard views in TTE. Five online resources flanked the 120- min front-of-class lecture according to Fig. 1. The participants received an email inviting them to take part in the first two online resources. The first one was a hose figure test on spatial orientation [18] (online supplement). Five tasks evaluated the spatial orientation capacity of the participants. Jars with tubes in them were photographed from two sides; the first picture showed the front view. The second showed either the left side, the right side, or the rear side. The students’ task was to choose the correct view.

The second online resource gathered baseline-information: participation in previous sonography courses, being a tutor-student in sonography, and previously visited preclinical classes on cardiac anatomy. Additionally, a five-point Likert scale recorded their self-assessment concerning their experience in sonography, echocardiography, and cardiac anatomy.

**Fig. 2:**
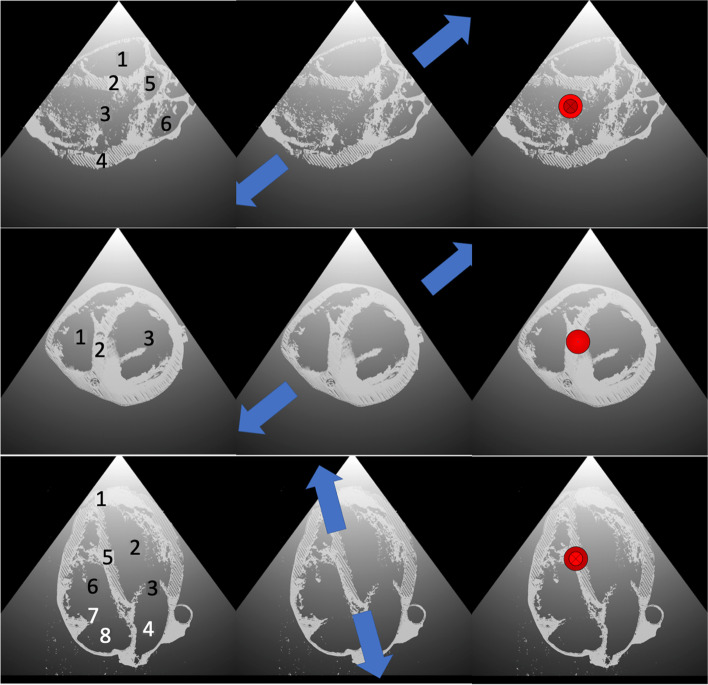
Nine test images. Each row shows PLAX, PSAX, and A4C views. The first row asks for naming the numbered structures. The second row asks for extremities, the arrows point on. I.e. center top image: upper right arrow: right shoulder. Lower left arrow left hip. Center middle image: upper right arrow: left shoulder. Lower left arrow: right hip. Center bottom image: upper arrow (apex) left hip. Lower arrow (base): right shoulder. Right row: Top image: The arrow is pointing into the PLAX plane, therefore it points towards the right hip. Middle image: the arrow is pointing out of the PSAX plane, therefore pointing towards the left hip. Bottom image: The arrow is pointing into the A4C plane, therefore pointing to the anterior chest wall

The students participated in the 120-min echocardiography class within the adjacent week. Groups of up to eight students started the class with the third online resource called the ‘ex-ante’ pre-test. The test showed artificial pictures of three selected echocardiographic standard views (Fig. 2) and nine questions. In the first three questions, the participants described numbered, anatomical structures. In the second three questions, the participants had to point out what extremities the blue arrows in the 2D plane would point on. In the last three questions, the participants had to name what anatomical structure a red arrow pointing 90° in or out of the picture would most likely point on. They could reach a total score of 41 points. One point was awarded for each correct description of an anatomical structure (total 20 points. Figure 2 left row), 2 points for a right answer of a 2D direction (total 12 points. Centre row), and 3 points for a correct guess of a 3D direction (total 9 points. Right row). The complete test and answers are displayed in the online supplements. After the pre-test, the tutor revealed the answers for the anatomy naming exercise.

### Design of test-questions

The questionary, formally consisted of the subdomains factual knowledge (anatomy), 2D orientation, 3D orientation, and each domain was represented by multiple questions. The questions demanded free-text answers for more discriminative power than multiple-choice questions.

We used open questions demanding free text answers due to the reasons described by Loke et al. [10]. Open questions have the advantage that the students cannot guess from multiple choices but must find their own answers. Thus, more in-depth insight into the students’ solution production process was possible, guessing was eliminated, and a correct answer had much more validity.

### Teaching and training intervention

Voluntary pairs examined each other with the four echocardiography devices (ACUSON X 300 PE, Version 7.0, Siemens Healthcare, Erlangen, Germany. Innosight, Philips Ultrasound Inc. Bothell, WA, USA). The tutor occasionally guided and corrected each student pair until they could reproduce the three standard views: apical four-chamber view (A4C), parasternal long-axis view (PLAX), and parasternal short-axis view (PSAX). Thereafter, the participants heard a short lecture on common echocardiographic findings in emergency medicine. Then the participants performed the identical retest. Finally, they filled in an online questionnaire, asking them about their perception on the helpfulness of teaching means and to rate the difficulty of solving the tests.

### 3D-printed models

**Fig. 3:**
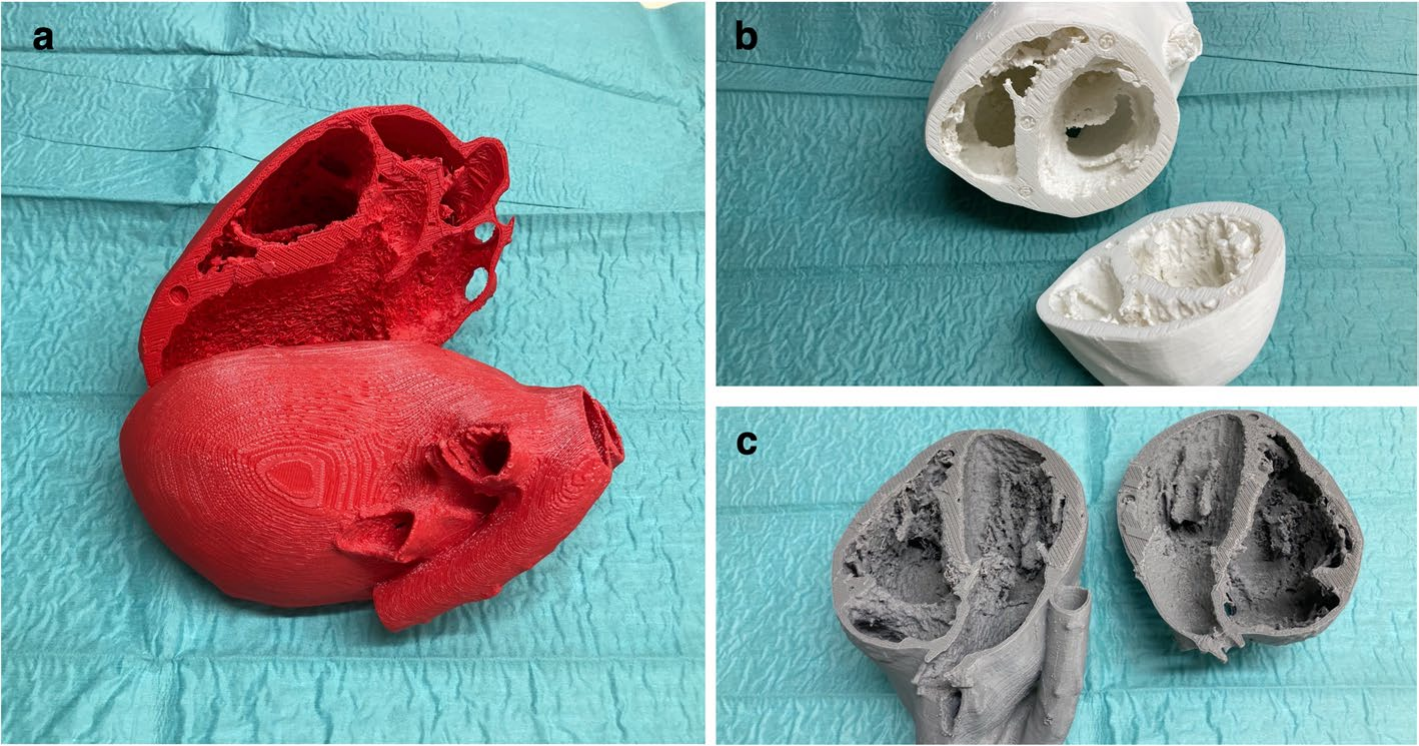
Three 3D printed cardiac models resembling parasternal long axis view PLAX (**a**), parasternal short axis view (**b**), and apical four chamber view (**c**). The trainee may orientate the model according to the echocardiographic view and resolve, which part of the model is “cut away” and which part remains

The 3D printing files were retrieved from the Resuscitative TEE Project website (https://www.resuscitativetee.com/3d-printed-models) (Fig. 3). Strictly speaking, these models are taken from the transesophageal point of view, not from the transthoracic one. They include the mid esophageal long-axis view, trans gastric short-axis view, and mid-esophageal four-chamber view. These nuances were neglectable for the level of understanding necessary for this TTE class. The printing files were fed to a MakerBot replicator 3D filament printer (MakerBot Industries, Brooklyn, NY, USA). The fused deposition modelling filament was an economic standard polyacrylic acid. The printing took approximately 12 h per item. The heart models are detachable at exactly the planes of the echo standard views.

### Intervention

One hundred ninety-five students were randomly assigned to one of 24 groups over 12 weeks. The first 12 groups were taught with the use of 3D printed models of the heart. The models were available at any time after the pre-test, even during the retest for the students’ haptic impression and spatial orientation. In the control group, the tutor used only slides to present the standard views. The 3D models were not brought in until the retest answers were provided.

### Statistics

We aimed to include half the students in the 3D model intervention group and half in the control group. All available cases were analyzed with SPSS 25 (IBM Corp., Armonk, NY, USA). Continuous variables were tested for normal distribution by the Shapiro-Wilk test. Normally distributed data are reported as means and standard deviations (SD); otherwise, the medians, quartiles and ranges are reported. Categorical data are reported as counts and percentages. For inferential statistical comparisons of categorical variables, the *χ*^2^ test was used. For continuous variables, the Mann-Whitney U-test for independent samples was used when the variable is not normally distributed and Student’s t-test for unpaired samples was used when the variable is normally distributed. A Wilcoxon-test analyzed pre-test-retest comparisons. The change of abilities was calculated by subtracting the pre-test score from the retest score (depicted in the online supplement). The distribution of the individual differences between pre- and retest scores in the control and intervention groups fulfilled criteria of normality for the overall test results but not concerning the subdomains (factual knowledge, 2D orientation, 3D orientation) of the test. We performed a multivariate linear regression with ‘improvement in the visual thinking score’ as the dependent variable. The visual thinking score comprises the subdomains 2D and 3D orientation and was formed by adding the respective points from the questionnaire. The independent variables were ‘age’, ‘gender’, ‘3D model intervention’, ‘abdominal sonography class’, ‘score in the hose-figure test’, and ‘visual thinking pre-test score’. These potential explanatory variables were chosen based on expert opinion and reasoning without statistical preselection. An analysis of variance (ANOVA) was carried out for self-assessment and test results. Only complete data sets were admitted for statistical analysis. The evaluation strategy was supported by the Institute of Clinical Epidemiology and Applied Biometry of the University of Tübingen. Statistical analysis and data reporting complied with the EACTS statistical and data reporting guidelines [6].

## Results

### Baseline parameters

From the 195 students evaluated, 164 gave written informed consent. The others attended the class, but no data was recorded. 153 (100%) students returned valid results (11 datasets had to be excluded due to incomplete answers). 89 (58.17%) students were female. The mean age was 25.9 +/− 2.9 years. Experience in sonography was as follows: 72.5% (111) attended the abdominal sonography class, 9.2% (14) were peer-teaching tutors, 1.96% (3) attended the preclinical heart study group, and 11.1% (17) had clinical experience. Just 12.4% (19) reported having no practical experience. On a Likert-scale from 1 (poor) to 5 (excellent) points, the participants had to self-assess their expertise before the class (Table 1).

**Table 1:** Self-assessment of knowledge and experience in sonography, echocardiography, and cardiac anatomy of 153 participating students. The majority of the students state fair expertise in sonography, poor to little expertise in echocardiography, but fair to good knowledge in cardiac anatomy

Self-assessment	poor	little	fair	good	excellent
(*n* = 153)					
**Sonography**	13 (8.5%)	38 (24.8%)	61 (39.9%)	36 (23.5%)	5 (3.3%)
**Echocardiography**	58 (37.9%)	50 (32.7%)	27 (17.7%)	17 (11.1%)	1 (0.7%)
**Cardiac anatomy**	2 (1.3%)	25 (16.3%)	66 (43.1%)	57 (37.3%)	3 (2.0%)

The control group (*n* = 76) and the intervention group (*n* = 77) did not differ significantly with respect to age, gender, experience, and self-assessment. The majority of the control group patients (67/76, 88%) had already participated in an abdomen-sonography class, whereas this was only true for 44/77 (57%) of the patients in the intervention group (Table 2). There were no significant differences between groups in the pre-test scores (total- *p* = 0.582, anatomy- *p* = 0.238, 2D- *p* = 0.929, 3D- *p* = 0.083, visual thinking score *p* = 0.274).

**Table 2:** Characteristics of the study groups. There were no significant differences regarding age, gender, semester, self assessment of sonography, echocardiography, and cardiac anatomy

Quality	Control Group	3D Group	***p*** value
number of participants	76	77	
age years	25.97	25.78	0.682*
gender (women)	39	50	0.088^+^
sonography class	67	44	0.000^+^
peer teacher	8	6	0.558^−^
heart class	2	1	0.620^−^
self-assessment	poor some fair good excellent		
in sonography	8 16 31 18 3	5 22 30 18 2	0.763^+^
in echocardiography	26 31 13 5 1	32 19 14 12 0	0.094^+^
in cardiac anatomy	1 16 32 25 2	1 9 34 32 1	0.491^+^

^*^t-Test

^+^Chi2-Test

^−^Fisher’s exact test

### Effect of the teaching intervention

**Fig. 4:**
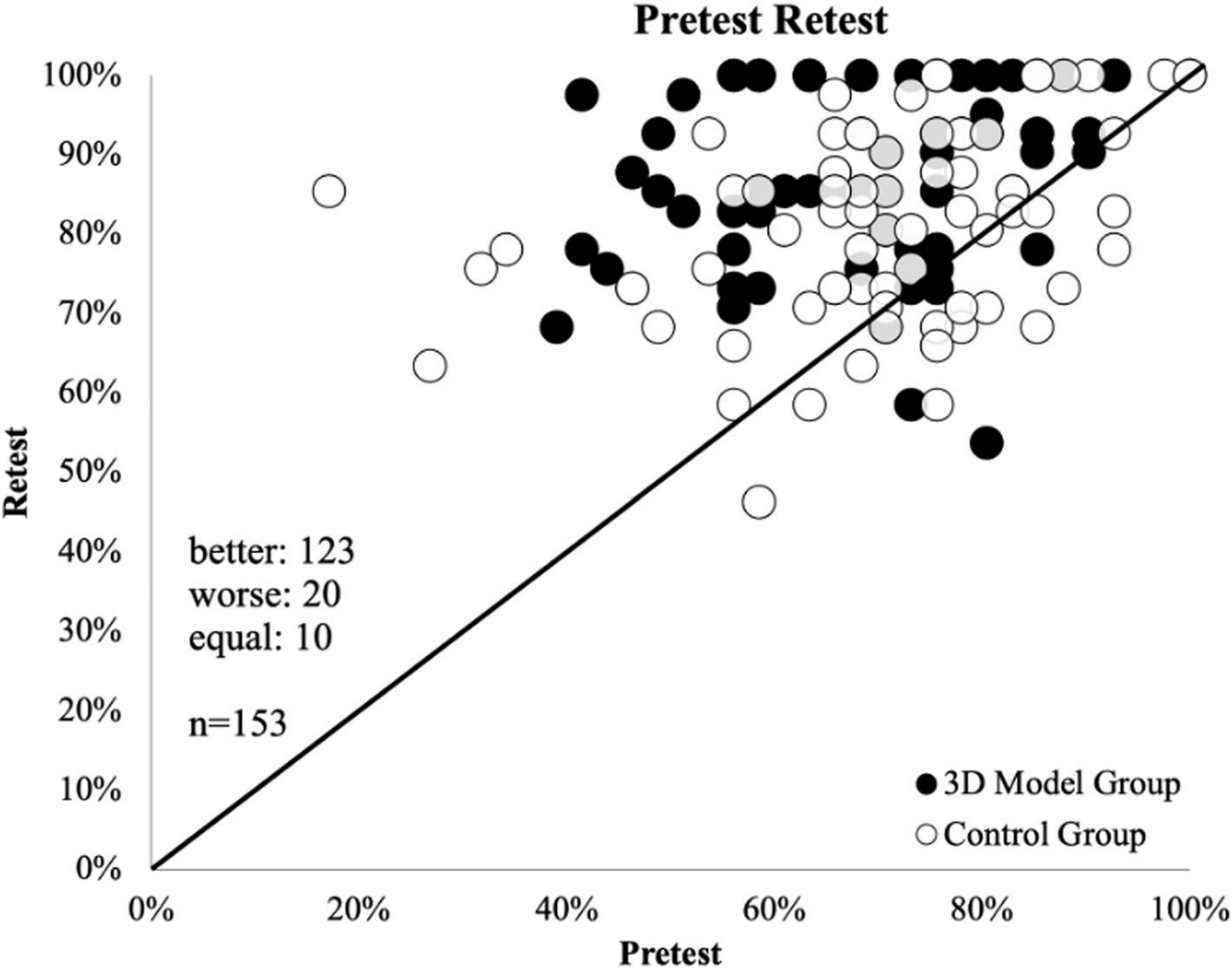
Pre-test-Retest-Diagram: The x-Axis depicts the pre-test-Score, the y-Axis depicts the retest-Score. Of 153 participants, 123 scored better, ten scored equal, and 20 worse in the retest than in the pre-test

**Fig. 5:**
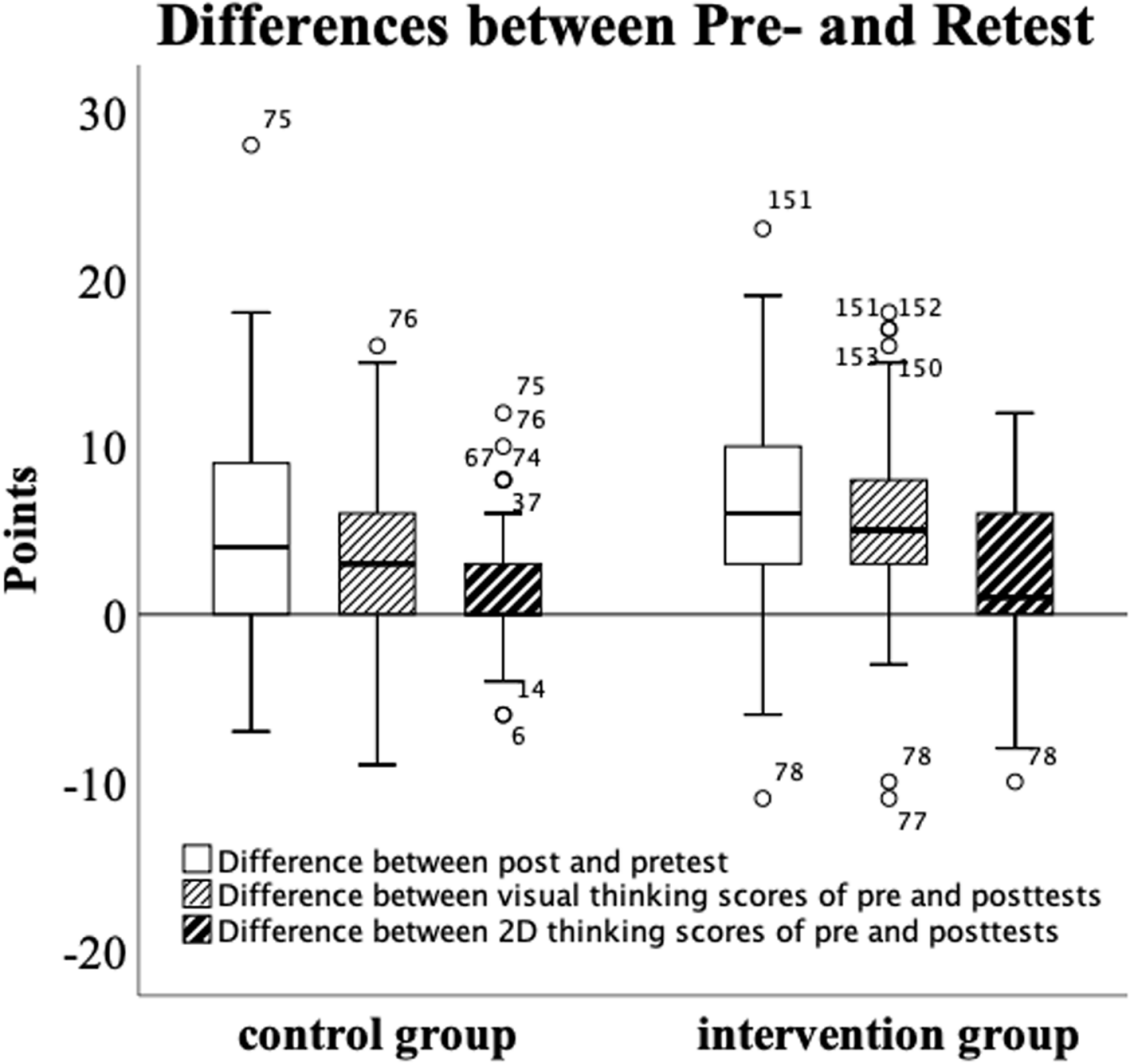
Boxplot of the Score Differences regarding the control and 3D intervention group. The visual thinking score differs most

The intraindividual changes are depicted in Fig. 4. Of 153 students, 123 improved their test results, 20 did worse, and ten achieved the same result. The median overall pre-test score was 29 of 41 points, and the median retest score was 35 (*p* < 0.001). Both groups improved significantly. In the control group, subjects improved by a mean of 4.6 ± 6.5 points. In the 3D model intervention group, the mean improvement was significantly greater (7.0 ± 6.0 points, *p* = 0.016) (Fig. 5).

Upon considering the subdomains, we found a significant improvement in the intervention group compared with the control group with respect to 2D-orientation (*p* = 0.002) and visual thinking (*p* = 0.006), and a strong tendency with respect to 3D orientation (*p* = 0.088) (Fig. 5).

A qualitative observation from the course: Students claimed the models largely contributed to their understanding. In addition, they often requested them for their learning sessions and gave positive reviews.

### Predictors of post-test visual thinking

A backward multivariate linear regression model sought to identify independent variables that predict an improvement from pre- to retest. From the candidate independent variables age and gender were eliminated as irrelevant. Likewise, the test score in the hose-figure test was not predictive (Table 3). Predictive variables were teaching with 3D models (β_1_ 2.96, CI 95% 1.487–4.44, *p* < 0.001), a good visual thinking pre-test score (β_2_–0.704, CI 95% - 0.856 – - 0.551, *p* < 0.001), and participation in the concomitant abdominal sonography class (β_3_ 1.994, CI 95% 0.347–3.642, *p* = 0.018). The model achieved a corrected R^2^ of 0.404. The effect size according to Cohen was f^2^ = 0.62. Importantly, teaching with the 3D models revealed to have the strongest influence on the increment of post-test visual thinking. The students, on average, scored 2.96 points more when being taught using the 3D models.

**Table 3:** Regression model. Model 1 includes all the relevant variables based on expert’s opinion. Model 2–4 exclude variables in a backward regression model according to *p* values > 0.1. Gender, age, and hose figure test were excluded from the model. The VT pre-test score, the sonography course, and the 3D model remained in the regression model

Model	Included variable	Excluded variable	Method
1	Pre-test VT score Age Sonography course Hose figure test Gender 3D model	–	Inclusion
2	–	Gender	Backwards, p of F > =0.1
3	–	Age	Backwards, p of F > =0.1
4	–	Hose figure test	Backwards, p of F > =0.1

### Self-assessment and pre-test results

The students completed a self-assessment regarding the difficulty of the questions in the pre-test with a 5-point Likert scale. Later the 5 point scale was aggregated into three categories (1–2 easy, 3 medium, 4–5 difficult) and compared with the objective test results. Students with subjective difficulty in the hose figure test, objectively scored only 75% (CI 95% 63.25–86.75) whereas students rating it easy scored 92% (CI 95% 88.02–94.71, *p* = 0.001). The same effect was present for anatomy questions (17.61 pts. vs. 18.66 pts.; *p* = 0.003) and 2D-thinking questions (7.47 pts. vs. 10.11 pts.; *p* = 0.003), respectively. In summary, in all three questionnaire categories, student self-assessment was related to performance. This was not the case for performance in 3D-thinking questions.

### Teaching methods

The assessment of the helpfulness of certain teaching methods had a ceiling effect: as 5 points could be given as a maximum, most students gave 5 points for practicing, tutor explanation, and 3D models. Slides of the standard views were rated lower. For difficulty, students rated the hose figures easiest followed by the anatomical descriptions. 2D questions were rated difficult to very difficult. The 3D questions were rated very to most difficult. (see online supplementary material).

## Discussion

Anatomical models are traditionally available for a more in-depth understanding of the topology of organs, and haptic models transport this concept better than 2D images or virtual reality computer models [7, 16]. 3D printed models have been used to teach anatomical structures with good reception [4, 11], and even specific pathologies can be depicted visually and haptically in high resolution [10].

TTE is not part of the core curriculum of medical students in Germany, but it is suggested by the national competence based catalogue of learning objectives in medicine [13]. Echocardiography is also part of the training of residents in cardiac surgery and cardiology. Our didactic concept appears to be transferrable into this context and we discussed to conduct a study with residents as subjects. However, such a study was hampered by methodological obstacles: Due to the limited number of residents and their interindividually different levels of knowledge, neither the number of cases nor the comparability that would have been required for a study could be achieved, at least in the monocentric design. When confronted with TTE, students often struggle with correlating the 2D picture displayed with the 3D anatomy. This difficulty could be explained by the predefined TTE standard views (PLAX, PSAX, A4C) not correlating with the anatomical illustrations (transversal, frontal and sagittal planes). Therefore, in 2017 we introduced the detachable 3D printed cardiac models in our voluntary TTE-class, providing a tool for the students to apply their anatomical understanding to the echocardiographic picture. Due to the Covid-19 restrictions of bedside teaching in 2020, our previously voluntary echo curriculum [2] became obligatory for an entire semester cohort under lab conditions. This gave us the opportunity to cross-sectionally teach and study an entire unselected semester cohort without the selection bias of voluntary programs which are otherwise mostly selected on the basis of personal interests and talents. Earlier, the Resuscitative TEE Project published their findings on various aspects of transesophageal echocardiography in emergencies [3, 8, 19–21]. Among their resources, data sets for 3D printable models are available. These models are essential to this study. McKenna et al. described a 3D printed trainer for the in vitro training of TEE; here, the focus was the phantom model and not the cardiac model itself [12]. Ochoa et al. also used 3D models in TTE teaching and could not detect a significant effect on learning efficiency, which somehow is contradictory to our results but is probably secondary to the smaller group size and the different setup of their study [14]. This underlines the need for larger-scale, in depth studies on the role of three-dimensional models in evidence-based medical- and surgical education.

The central findings of this study are: (a) the intervention group taught with the use of 3D models scored significantly better overall, and in terms of visual thinking, and (b) the 3D models have the most substantial positive influence (effect size) on the post-test results.

Self-assessment and test scores showed that a higher perception of difficulty coincided with a lower test score, which is conclusive. Especially students with below-average pre-test results in visual thinking benefit considerably from the 3D models. Students rated the learning experience very positively. Thus, teaching with haptic 3D-models subjectively and objectively leads to more effective teaching and learning in echocardiography classes.

With this, we provide evidence for the superiority of the use of three-dimensional models compared to conventional imaging-based teaching materials in echocardiographic training and strengthen the basis for further systematic teaching research in the field. The strategy may be transferred to other sonography classes, especially given the fact that the 3D printing technology enables creating models tailored to the suits of the students and according to the didactic requirements in a cost effective and timely manner.

### Study limitations

There were no formal sample size calculation prior to the study, we cross-sectionally analyzed an entire unselected semester cohort instead. The number of test-questions to assess each level of understanding (factual knowledge, 2D thinking, and 3D spatial orientation) was limited to three each, which might be discussed as relatively few, and the teaching intervention itself was restricted to a one-day session, which is critically short. On the other hand, the relatively large number of participants may compensate for some of these shortcomings.

In the same semester, our colleagues from internal medicine performed an abdominal sonography class for the same cohort [2]. To restrict the possible bias from that course on our control rather than on our intervention group, we purposely introduced the 3D models in the first 12 of 24 groups, so that most students have not had the abdominal sonography course at that timepoint. Thus, the concurrent sonography class potentially lifted the visual thinking score of the control group, but not of the intervention group and could not have boosted the effect of the 3D models. Consequently, this bias potentially leads to underestimation but excludes overestimation of the teaching effect with the 3D-models.

Otherwise, the baseline characteristics of the groups did not differ (structural equality of the groups). The three-dimensional anatomical orientation of the echo views was subject of pre- and retests. This strategy of using the same test twice contains a learning bias, which is controlled by exposing both groups to that strategy. Larger longitudinal studies are warranted to compensate for the aforementioned limitations.

## Conclusion

The implementation of 3D printed models of the standard echocardiographic views PSAX, PLAX, and A4C lead to a significantly more effective training in echocardiography, especially regarding the transfer of visual-thinking-skills. Particularly students with below-average performance in visual thinking benefited from this intervention. This effect may be transferable to other teaching interventions and must be object to future studies in evidence-based medical- and surgical education.

## Data Availability

Data were recorded with the educational ILIAS system of the Medical Faculty of the University of Tübingen, Germany. Data were exported to and further processed with Microsoft Excel. Data analysis was carried out with IBM SPSS Version 25. For review purposes, all demanded data will be provided by the corresponding author on reasonable request.

## Abbreviations

2D: Two dimensional
3D: Three dimensional
TEE: Transesophageal echocardiography
TTE: Transthoracic echocardiography
PLAX: Parasternal long axis
PSAX: Parasternal short axis
A4C: Apical four chamber
NKLM: (German) national competence based catalogue of learning objectives
CME: Continuing medical education
COVID: Corona Virus Disease
SPSS: Statistical Package for Social Sciences

## References

[1] Bui I, Bhattacharya A, Wong SH, Singh HR, Agarwal A (2021). Role of three-dimensional visualization modalities in medical education. Front Pediatr.

[2] Celebi N, Griewatz J, Malek NP, Krieg S, Kuehnl T, Muller R (2019). Development and implementation of a comprehensive ultrasound curriculum for undergraduate medical students - a feasibility study. BMC Med Educ.

[3] Fair J, Tonna J, Ockerse P, Galovic B, Youngquist S, McKellar SH (2016). Emergency physician-performed transesophageal echocardiography for extracorporeal life support vascular cannula placement. Am J Emerg Med.

[4] Fasel JHD, Aguiar D, Kiss-Bodolay D, Montet X, Kalangos A, Stimec BV (2015). Adapting anatomy teaching to surgical trends: a combination of classical dissection, medical imaging, and 3D-printing technologies. Surg Radiol Anat.

[5] Fehske W, Flachskampf F, Helfen A, Kreidel F, Kruck S, Rosée KL, et al. Manual zur Indikation und Durchführung der Echokardiographie – Update 2020 der Deutschen Gesellschaft für Kardiologie. Kardiologe. 2020;14:1–34.

[6] Hickey GL, Dunning J, Seifert B, Sodeck G, Carr MJ, Burger HU (2015). Statistical and data reporting guidelines for the European journal of cardio-thoracic surgery and the interactive CardioVascular and thoracic surgery. Eur J Cardiothorac Surg.

[7] Khot Z, Quinlan K, Norman GR, Wainman B (2013). The relative effectiveness of computer-based and traditional resources for education in anatomy. Anat Sci Educ.

[8] Lim KHA, Loo ZY, Goldie SJ, Adams JW, McMenamin PG (2015). Use of 3D printed models in medical education: a randomized control trial comparing 3D prints versus cadaveric materials for learning external cardiac anatomy.

[9] Link D, Albers J, Vahl CF (2014). Basic transthoracic echocardiographic skills in cardiac surgery. Zeitschrift Herz Thorax Gefäßchirurgie.

[10] Loke Y-H, Harahsheh AS, Krieger A, Olivieri LJ (2017). Usage of 3D models of tetralogy of Fallot for medical education: impact on learning congenital heart disease. BMC Med Educ.

[11] Lozano MTU, Haro FB, Ruggiero A, Manzoor S, Méndez JAJ (2019). Evaluation of the applicability of 3d models as perceived by the students of health sciences. J Med Syst.

[12] McKenna RT, Dove JC, Ratzlaff RA, Diaz-Gomez JL, Cox DJ, Simon LV (2018). A 3-dimensional printed ultrasound probe Visuospatial trainer. Ultrasound Q.

[13] Medizinischer Fakultätentag National Competence Based Catalogue of Learning Objectives for Undergraduate Medical Education, Nationaler Kompetenzbasierter Lernzielkatalog Medizin, NKLM. 2015. http://www.nklm.de/files/nklm_final_2015-07-03.pdf. Accessed 8 Feb 2021.

[14] Ochoa S, Segal J, Garcia N, Fischer EA (2018). Three-dimensional printed cardiac models for focused cardiac ultrasound instruction. J Ultrasound Med.

[15] Ochoa S, Segal J, Garcia N, Fischer EA (2019). Three-dimensional printed cardiac models for focused cardiac ultrasound instruction. J Ultrasound Med.

[16] Silén C, Wirell S, Kvist J, Nylander E, Smedby O (2008). Advanced 3D visualization in student-centred medical education. Med Teach.

[17] Sodian R, Schmauss D, Markert M, Weber S, Nikolaou K, Haeberle S (2008). Three-dimensional printing creates models for surgical planning of aortic valve replacement after previous coronary bypass grafting.

[18] Stumpf H, Fay E. Schlauchfiguren. Göttingen: Hogrefe Verlag; 1983.

[19] Teran F (2019). Resuscitative cardiopulmonary ultrasound and transesophageal echocardiography in the emergency department. Emerg Med Clin North Am.

[20] Teran F, Dean AJ, Centeno C, Panebianco NL, Zeidan AJ, Chan W (2019). Evaluation of out-of-hospital cardiac arrest using transesophageal echocardiography in the emergency department. Resuscitation..

[21] Teran F, Prats MI, Nelson BP, Kessler R, Blaivas M, Peberdy MA (2020). Focused transesophageal echocardiography during cardiac arrest resuscitation: JACC review topic of the week. J Am Coll Cardiol.

